# Impact of Textile Industries on Surface Water Contamination by Sb and Other Potential Toxic Elements: A Case Study in Taihu Lake Basin, China

**DOI:** 10.3390/ijerph20043600

**Published:** 2023-02-17

**Authors:** Feipeng Li, Ziyi Guo, Lingchen Mao, Junyi Feng, Jiong Huang, Hong Tao

**Affiliations:** School of Environment and Architecture, University of Shanghai for Science and Technology, Shanghai 200093, China

**Keywords:** Sb, seasonal variation, source identification, factor analysis, pollution index, textile wastewater

## Abstract

Contamination of industry-derived antimony (Sb) is currently of great concern. This study was conducted to identify the source of Sb together with other potential toxic elements (PTEs) in a typical industrial area in China and emphasize the contribution of Sb to ecological risk in the local aquatic environment. By investigating the distribution of nine PTEs in surface water in Wujiang County in dry and wet seasons, this study revealed that textile wastewater was the main source of Sb. The distribution of Sb (0.48~21.4 μg/L) showed the least seasonal variation among the nine elements. Factor analysis revealed that the factor that controlled Sb distribution is unique. In general, Sb was more concentrated in the southeastern part of the study area where there was a large number of textile industries, and was affected by the specific conductivity and total dissolved solids in water (*p* < 0.01). Sb concentration in 35.71% of samples collected from the drainage outlet exceeded the standard limit of 10 μg/L. Results from three pollution assessment methods suggested that >5% of the sampling sites were slightly too heavily polluted and Sb contributed the most. Therefore, it is necessary to strengthen the administrative supervision of local textile enterprises and elevate the local standard of textile wastewater emission.

## 1. Introduction

Textile industries make a considerable contribution to the gross domestic product (GDP) and economic growth of many countries globally, especially in Asia, such as China, India, Pakistan, Bangladesh, and Malaysia, among others [1,2,3,4]. On the other hand, they may also bring considerable contamination to the local environment [5,6]. It is noteworthy that China has a long history of textile manufacture and has been ranked first all over the world with its annual production capacity of fabrics made by natural and synthetic fibers, such as cotton textiles, silk products, woolen fabrics, polyamide, nylon, and so on [7]. The Chinese textile industry is a significant section of the national economy and is mainly distributed in coastal regions, with more than 50% of textile and dyeing plants in China located in Jiangsu and Zhejiang province [8,9,10]. It has been estimated that the textile industry uses 50~150 L of water per kg of textile material for wet processing on average [11,12]. Such water consumption inevitably leads to the textile industry being one of the largest producers of wastewater [13]. The waste effluent from the textile industry is often highly polluted and contains various kinds of hazardous and refractory contaminants, mainly composed of acids, alkalis, dyes, toxic elements, and diversiform organic compounds [14]. Due to the needs of production, textile wastewater is continuously discharged. Contaminants in textile wastewater are directly or indirectly transferred into the aquatic environment, causing the risk of deteriorating the ecological environment continuously [3]. 

Early studies have reported that multiple potential toxic elements (PTEs) either in free ionic metals or complex metals [15] were detected in the river near textile factories and effluent of the textile industry worldwide, such as chromium (Cr), nickel (Ni), copper (Cu), zinc (Zn), cadmium (Cd), lead (Pb), cobalt (Co), antimony (Sb), arsenic (As), among others [1,16,17]. Exposure to PTEs lastingly doing great harm to human health has been proven by plenty of studies and Sb is considered a highly toxic contaminant due to its carcinogenic properties, damage to human immune and nervous systems, and severe ecological toxicities [18]. Though Sb residues in treated textile wastewater are presented at low concentration levels [19], the bioaccumulation arising from drinking water and consuming aquacultural fish containing Sb threatens human health, as Sb in mammals can- not be detoxified by methylation like As [20,21]. In past decades, a series of critical reviews on the occurrence of Sb have been published, arousing the scientific community and society more interest in Sb contamination over different environmental compartments [22]. Therefore, European Union, the United States, and China have set the drinking water standards with the maximum admissible concentration (MAC) of Sb as 5 μg/L, 6 μg/L, and 5 μg/L, respectively [17]. In 2012, China revised the standard on textile effluent emission by adding the MAC of total Sb concentration with 0.1 mg/L (GB 4287-2012).

Nevertheless, some of the previous studies focused on developing novel treatments to remove potential toxic elements, especially Sb, from the textile effluent by the synthetic compounds which can adsorb or complex metal cation [18,23,24]. Other studies paid more attention to the evaluation and prediction of potential ecological risk generated from Sb and other PTEs by understanding the speciation and migration of PTEs, which are generated from treatment plant effluent in the surface water or wetlands [25,26]. However, there is little knowledge about the relationship between textile industry distribution and the contribution to the distribution and ecological risk of PTEs, especially emerging contaminants of Sb, in surface water at a large region. 

Therefore, in this study, over one hundred sampling sites were set in the region of Wujiang County, Jiangsu Province, China, which is one of the important textile industry agglomerations at home [27]. The intensive textile industry and anthropogenic activities near the river network have brought a significant negative impact on the water quality in this region. The distribution of nine PTEs in the surface water of these sampling sites was determined to aim at (1) exploring the relationship between the source of PTEs, especially Sb, and local textile industry distribution, (2) discussing the relationship between water properties and PTE concentrations, (3) analyzing the potential eco-risk of PTEs on the local aquatic environment and Sb’s contribution on eco-risk so that further information for local administrative department managing the ecological environment to establish a more appropriate standard and administer the wastewater emission of local textile enterprises.

## 2. Materials and Methods

### 2.1. Study Area

Wujiang County in Jiangsu Province is one of the well-known textile industry centers in China, and it covers a plain area with complicated water network attached to Taihu Lake Basin. As a result of the flourishing textile industry, there are several industrial zones with a high density of textile plants in Wujiang. The total number of textile enterprises can reach hundreds, most of which are concentrated in the south of the Taipu River, and the treated wastewater from textile industries accounts for more than 95% of the total sewage discharged in the region [28]. Taipu River, one of the main streams that flow through Wujiang, is utilized as a channel to transport the fresh water from Taihu Lake to Jinze Reservoir which is the drinking water source for Shanghai [29]. Therefore, pollutants in textile wastewater will cause the risk of downstream drinking water safety and local aquatic environment protection.

**Figure 1:**
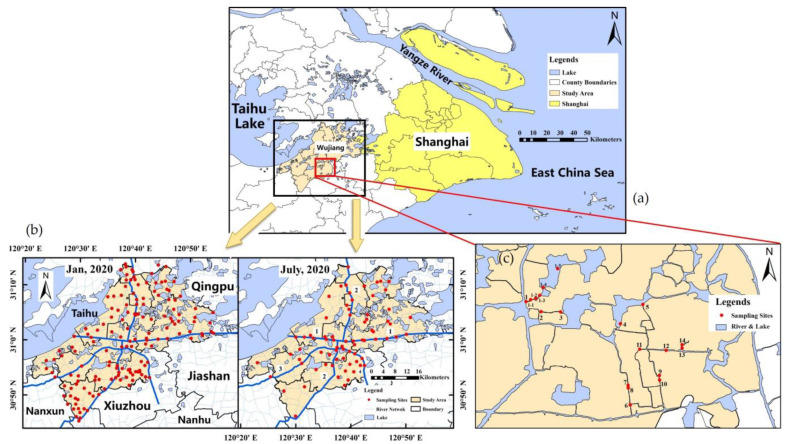
Map of study area (**a**), sampling sites distribution in Wujiang area. Water samples were collected in January 2020 (*n* = 154) and July 2020 (*n* = 74) (**b**) and sampling sites (*n* = 14) at drainage outlets in January 2020 (**c**).

The target study area, Wujiang County, is located in the southern part of Jiangsu Province and adjacent to Shanghai City, covering an area of 1176 km^2^. The study area lies between 30°45′36″–31°13′41″ N and 120°21′04″–120°53′59″ E (Figure 1a), and the river network area as well as more than three hundred lakes of various sizes within the boundary of Wujiang belong to Taihu Lake Basin. The main streams in the river network area are Taipu River, Jinghang Canal, Ditang River, and Sujia Canal. Plenty of tributaries of four main streams which connect to each other were distributed intensively in the study area with river density reaching 3.25 km/km^2^. The surface water area covers 270 km^2^ approximately, accounting for 22.7% of the total area of Wujiang County, which suggests the study area is a typical intensive region of water network. This kind of geohydrological feature results in the fact that the flourishing agriculture and aquaculture, a large number of older areas as well a relatively dense distribution of residential neighborhoods and industrial zones exist in the urban and rural areas of Wujiang County. 

### 2.2. Sampling and Experimental Methods

To study the spatial and temporal changes in the potential toxic element concentrations in the surface water during dry period and wet period, the surface water samples at 154 and 74 sampling sites located in the region which had a high density of water network in study area were collected in January (dry period) and July (wet period), respectively, based on our previous investigation about the distribution of industrial enterprises involved in wastewater emission and the area where these enterprises are located (Figure 1b). Moreover, 14 water samples at the sites adjacent to drainage outlets of textile enterprises were collected in January 2020 (Figure 1c). Among these sites, 5 downstream samples near site 1, which was a drainage of a textile wastewater treatment plant, were collected to further discuss the PTEs contents in surface water. The distribution of sampling sites was shown in Figure 1, and all sampling sites were positioned by Ovital Map (Yuanshenghua Ltd., Beijing, China). The water samples were collected from the rivers at a depth of 0.3~0.5 m below the surface. Each sample with 500 mL volume was stored in a pre-cleaned polyethylene bottle after acidification to pH < 4 with diluted HNO_3_, and then transported back to the laboratory as soon as possible. The samples were stored in the refrigerator at 4 °C until being pretreated, and they were filtered through 0.45 μm membrane filters before analysis. 

### 2.3. Water Analysis

Water temperature, pH, DO (dissolved oxygen), SPC (specific conductivity), TDS (total dissolved solids), turbidity, COD (chemical oxygen demand), fDOM (fluorescent dissolved organic matter) and Chl (chlorophyll) of each sampling site were in situ monitored employing YSI EXO Ⅱ (Xylem Corporation, WhitePlains, NY, USA). The water samples for PTEs measurement were pretreated as the Chinese standard of HJ 677-2013. Fifty milliliters of each sample were taken into columned Teflon tube with the cap, and five milliliters of HNO_3_, as well as three milliliters of H_2_O_2_, were added to the sample in sequence. Then, the capped tube with mixed solution was digested on the electric heating plate, and digestion was completed within 30 min. The remained solution was transferred into 50 mL volumetric flask, and the volume was made equal to 50 mL using distilled water. Finally, concentrations of 9 PTEs in the surface water samples including Cr, Ni, Cu, Zn, Cd, Pb, Co, As, and Sb were determined by using inductively coupled plasma mass spectrometry (ICP-MS, NexION 300X, PerkinElmer Corporation, Mass, MA, USA). All reagents employed in this study were at the grade of GR (guaranteed reagent) or AR (analytical reagent), and all plastic and glass containers were acid washed by soaking in diluted HNO_3_ for at least 24 h.

### 2.4. Assessment of Water Quality and Ecological Risk

Three methods including the heavy metal pollution index (*HPI*), the pollution load index (*PLI*), and Nemerow pollution index (*NPI*) were used to evaluate water quality and ecological risk in the water. 

The index of HPI is a rating model that represents the composite influence of PTEs on the overall quality of water [30]. The formula of HPI initially introduced in 1996 [31] is composed of the unit weight (*W_i_*) and the sub-index (*Q_i_*). The *W_i_* values are made to be inversely proportional to the recommended standard for the corresponding parameter in order to reflect the relative importance of the potential toxic element parameters [32,33]. However, unlike metals in soil or sediments, for the reason of the migration of PTEs in the upper water susceptible to be affected by hydraulic disturbance, no background reference value is available for the upper water of rivers and lakes. Therefore, the national standard limit of potential toxic element contents in Class Ⅲ surface water (GB 3838-2002, China) was taken as the background value, and the *HPI* model was calculated as follows:

(1) $$HPI=\frac{\sum_{i=1}^{n}\left(W_{i}{\times \;Q}_{i}\right)}{\sum_{i=1}^{n}W_{i}}$$

where *Q_i_* is the sub-index of the *i*th PTE, *W_i_* is the unit weight of the *i*th PTE, and *n* is the number of parameters considered. The *Q_i_* is calculated as follows:

(2) $$Q_{i}=\frac{{MC}_{i}}{{SC}_{i}}\times 100$$

where *MC_i_* is the measured concentration of the *i*th potential toxic element parameter (μg/L), and *SC_i_* is the Class Ⅲ surface water threshold value of the *i*th potential toxic element parameter in the surface water specified in GB 3838-2002 for Cr, Ni, Cu, Zn, Cd, Pb, Co, As, and Sb, which are 0.05, 0.02, 1.00, 1.00, 0.005, 0.05, 1.00, 0.05, and 0.005 mg/L, respectively. The *W_i_* of the parameter is calculated as follows:

(3) $$W_{i}=\frac{k}{{SC}_{i}}$$

where *k* is a proportionally constant. The constant *k* is set to 1 for facilitating calculation. The *HPI* grading standard divided into four classes was shown in Table S1.

The *PLI* is defined as the *nth* root of the product of the contamination factors (*CFs*) of potential toxic element parameters, namely the geometric mean of the *CFs* [21], in order to determine the cumulative pollution load [34], and the *CF* values were the ratios of the detected values to the reference values. If *PLI* value is over 1, it means potential toxic element accumulation has occurred in the sampling site [35], posing potential risk to the surrounding ecological environment (Table S1). The *PLI* is calculated as follows:

(4) $$PLI={\left(CF_{1}\times CF_{2}\times \ldots \times CF_{n}\right)}^{1/n}$$

(5) $$CF_{i}=\frac{MC_{i}}{SC_{i}}$$

where *CF_i_* is the contamination factor of the *i*th PTE and *n* represents the number of PTEs.

Proposing initially by the American scholar N. L. Nemerow [36], the *NPI* is a quality index of multi-factors weighting that takes into consideration the mean and maximum value of *CFs* for the purpose of reflecting the pollution degree of the surface water at sampling sites comprehensively [37]. *NPI* is calculated as follows:

(6) $$\;NPI=\sqrt{\frac{{{(CF}_{\max})}^{2}{+(CF}_{ave}{)}^{2}}{2}}\;$$

(7) $${CF}_{\max}=\max(\;\frac{{MC}_{i}}{{SC}_{i}}\;)\;$$

(8) $${CF}_{ave}=\frac{1}{n}\sum_{i=1}^{n}\frac{{Mc}_{i}}{{Sc}_{i}}$$

where *CF_max_* and *CF_ave_* represent the maximum value and mean of *CFs*, respectively; and *n* represents the number of PTEs. The *NPI* values were categorized into four classes shown in Table S1.

### 2.5. Statistical Analysis

Study area and spatial distribution of the sampling sites were drawn using geographic information system software (ArcGIS 10.6, ESRI Inc., Redlands, CA, USA). The spatial and temporal distribution of potential toxic elements concentration was also drawn by ArcGIS using the spatial analysis of the inverse distance interpolation. The statistical analysis of physicochemical properties of surface water and potential toxic element concentrations was performed in Excel 2019 (Microsoft Corporation, Redmond, WA, USA), SPSS 26.0 (IBM Corporation, Armonk, NY, USA), and Minitab 20 (Minitab Inc., State College, PA, USA). Figures of risk assessment and horizontal dilution of Sb were plotted using Origin 2019b (OriginLab Corporation, Northampton, MA, USA).

### 2.6. Quality Assurance and Control

Before digestion, the mixed standard solution contained target PTEs (N9301721, PerkinElmer Corporation, Waltham, MA, USA) was diluted to a certain content to check the recoveries of PTEs in water and control the quality of digestion process. Recoveries of all target elements were in the range of 85–110%. Two blank samples and three standard samples were set for each batch of samples in the entire pretreating process. Meanwhile, one duplicate sample was set for every 5 samples in each batch, with all of the relative errors below 9%. The ICP-MS was set to read every sample i for three times and the results showed all RSDs were below 5% in the long-term run (<6 h). Additionally, Ge (10 μg/L), In (5 μg/L) and Bi (5 μg/L) were selected as the elements in internal standards to promote the accuracy of quantitative analysis of ICP-MS (HJ 400-2017, China). Ge was used to calibrate As, Cr, Cu, Ni, Co, and Zn. In was used to calibrate Cd and Sb, while Bi was used to calibrate Pb.

## 3. Results

### 3.1. Physicochemical Properties of Surface Water Samples

The results of the physicochemical properties of water samples collected in two seasons were shown in Table 1. The average values of surface water temperature, pH, DO, SPC, TDS, turbidity, COD, fDOM, and Chl were 8.81 °C, 7.43, 9.09 mg/L, 566.19 μs/cm, 370.87 mg/L, 53.79, 19.89 mg/L, 102.82, 7.02 μg/L in January, while those in July were 28.57 °C, 7.05, 6.48 mg/L, 387.40 μs/cm, 251.82 mg/L, 18.45, 31.66 mg/L, 11.66, 11.83 μg/L. The contents of Chl had the highest CVs in both months among these indicators. 

By comparing the means of these indicators between the two seasons, there was a significant decline in SPC, TDS, turbidity, and fDOM in July, suggesting a large amount of rainfall and increased volume of surface water during the wet period diluted the concentration of both dissolved and suspended matters in water. The higher value of COD in July implied an increase in organic matter which demand oxygen and brought down DO in water. In summer, the surface water in this area suffered from algal bloom (ref.) which led to a higher value of Chl. The coefficient of variation (CV) of pH values, DO, SPC, and TDS increased in July, reflecting that the special variability of water quality, especially the dissolved matter content in surface water, was significantly influenced by nearby human activities and hydrological conditions.

### 3.2. Spatial and Temporal Distribution of the Nine PTEs in Surface Water

Table 2 summarizes the PTEs concentration in surface water in both dry and wet seasons. The average concentrations of Cr, Ni, Cu, Cu, Cd, Pb, Co, As, and Sb were 1.17, 2.89, 5.78, 0.10, 2.42, 0.52, 1.65, 2.26 μg/L in January with the following sequence: Zn > Cu > Ni > Pb > Sb > As > Cr > Co > Cd; while those in July were 0.82, 3.04, 2.70, 0.08, 1.49, 0.48, 2.43, 1.48, respectively, as the following order: Zn > Ni > Cu > As > Pb > Sb > Cr > Co > Cd. Compared with the standard limits of Class Ⅲ surface water listed in GB 3838-2002, there was more PTEs contamination in the dry season than that in the wet season. In Jan, the concentration of Ni, Zn, and Sb in nine sampling sites exceeded the limited values, while only Sb concentrations in four sites exceeded the corresponding limit value in July. 

The variabilities of PTEs in January were all higher than that in July, i.e., higher CV. Fox example, in January, Zn had the highest average concentration of 24.68 μg/L with the highest CV of 433.74% and it similarly had the highest average concentration and CV of 27.69 μg/L, 110.86% in July, respectively. On the contrary, it has also been observed that only the absolute deviation Sb’s CVs (7.48%) in two months was below 10%, while the second lowest deviation of Co’s CVs was 5.32 times of Sb’s. To demonstrate and compare the content distribution in January and July more rigorously, the overall data were normalized into the same interval scale with [0,1]. Figure S1 compares the distribution of PTEs in Jan and July. It should be noticed that, although there was general difference in the level of Sb concentrations in two seasons present, the distributions of Sb were similar, i.e., the region with higher contents was mainly located in the southeastern part of study area. On the other hand, there was apparent regional discrepancy between the distribution of other PTEs in two seasons. Additionally, Cr and Cd contents were both too low to find the obvious difference.

### 3.3. Water Quality and Ecological Risk Assessment

In the light of aforementioned statistical analysis on PTEs contents, the proportion of the concentrations of Sb which exceeded the Class Ⅲ threshold in the surface water was obviously higher than that of other PTEs. Therefore, more information on the potential pollution risk of all target PTEs determined by three methods including *HPI*, *PLI*, and *NPI* (Table S1) was necessary to compare the contribution of Sb on pollution risk with that of other elements for the purpose of providing further information for the industrial wastewater emission management in this region.

**Figure 2:**
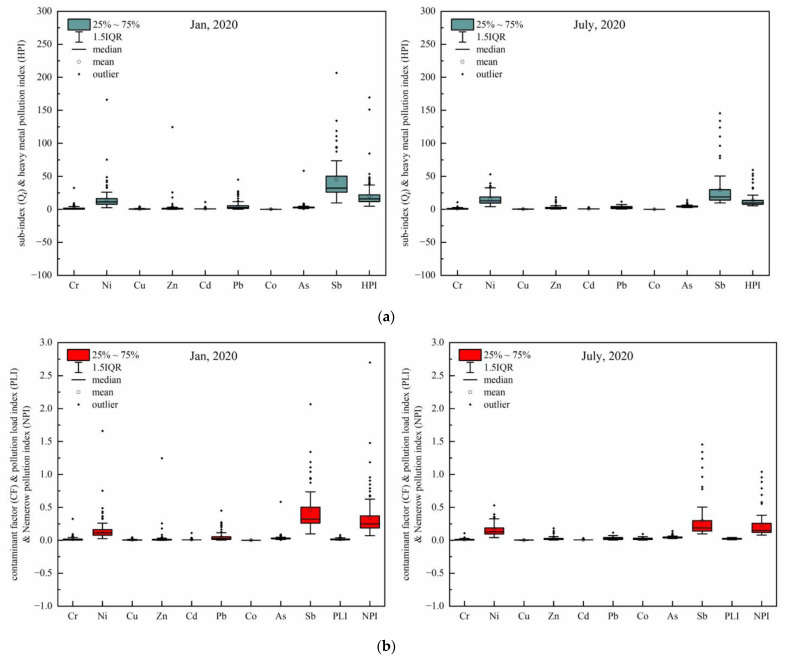
Box plots of the sub-index (*Qi*) and the heavy metal pollution index (*HPI*) values (**a**), the contamination factors (*CFs*), the pollution load index (*PLI*) and Nemerow pollution index (*NPI*) values (**b**) for all sampling sites in study area in January and July. The black dots are outliers.

The index of *HPI* was especially used to evaluate the potential pollution risk of heavy metals in water bodies. The *HPI* values of all sampling sites ranged from 4.71 to 169.51, with a mean of 19.79 in January; and they ranged from 5.44 to 59.67, with a mean of 13.67 in July. The *HPI* of all sampling sites in January, which was classified as slight, moderate, moderate-to-heavy, and heavy, accounted for 46.75%, 44.16%, 7.79%, and 1.30%, respectively, while that in July accounted for 79.73%, 10.81%, 0.00%, and 0.00%, respectively, according to *HPI* grading standards (Table S1). This suggests potential toxic element pollution risk classified as moderate in July was better than that in January. The distribution of all potential toxic element sub-indexes and *HPI* on the box plots was shown in Figure 2, demonstrating the numerical interval of Sb’s sub-index was larger than that of other PTEs.

To characterize the cumulative pollution, and load of target PTEs, the *PLI* and *NPI* were used, which were both calculated from *CFs* of trace elements. This method can help evaluate the effects of the combination of target PTEs on the water quality of the sampling site based on the standard limits. The distribution of *CF* for individual PTE was summarized in Figure 2. It is obvious that in both seasons, the *CF* of Sb was the highest among all PTEs with most of the sites ranging from 0.141 to 0.503, followed by that of Ni. The PLI of all sampling sites varied from 0.003 to 0.079 in January, and that in July varied from 0.010 to 0.044 (Figure 2). According to the classification of pollution level (Table S1), the results manifest that the overall effects of these PTEs on the water quality seemed to be negligible, but a small proportion of the *CF* of Sb, with 4.55% in January and 5.41% in July, was over 1 while almost all *CFs* of other PTEs were below 1, which means that the PLI cannot reflect the contribution of single *CF* on the index accurately under the circumstance that one single *CF* is much larger than other *CFs* that the other *CFs* are negligible.

To reflect the contribution of the largest single *CF* among all *CFs* more accurately, the *NPI* was used to take the mean of all *CFs* and the largest value of single *CF* into consideration and avoid the defect *PLI* has. The *NPI* of all sampling sites varied from 0.07 to 3.04 in January, and that in July varied from 0.08 to 1.04. The *NPI* of all sampling sites in January, which was classified as unpolluted, slight, moderate, and heavy accounted for 94.16%, 3.25%, 1.30%, and 1.30%, respectively, while that in July accounted for 94.59%, 4.05%, 1.35%, and 0.00%, respectively, according to *NPI* grading standards. 

### 3.4. Statistical Analysis

Statistical analysis was utilized to investigate the relationship between each PTE and water properties. Spearman correlation analysis among potential toxic element concentrations and a part of physicochemical indicators in the surface water of the study area were presented in Tables S2 and S3. Significantly positive correlations among PTEs found that there were 23 groups of 2 elements having positively significant cross-correlation at *p* < 0.01 and 2 groups at *p* < 0.05 in January, while 14 groups at *p* < 0.01 and 2 groups at *p* < 0.05 in July. In January, the highest cross-correlation existed in Cr and Ni (*r* = 0.798), while that existed in Ni and Zn (*r* = 0.739) in July. As for the physicochemical indicators, SPC and TDS were both positively significantly correlated with Ni and Sb at *p* < 0.01, Co at *p* < 0.05 in January, and with As and Sb at *p* < 0.01, Pb at *p* < 0.05 in July; turbidity was positively significant correlated with Cr, Ni, Zn, Pb, Co, As at *p* < 0.01 in January and with Cr, Pb, Co at *p* < 0.01, Ni at *p* < 0.05 in July; COD was positively significant correlated with As at *p* < 0.01 and Cd at *p* < 0.05 merely in July. 

To further analyze the relationship among these PTEs and the factors influencing their content, the factor analysis (FA) method was performed to obtain deep information on the data. The method of maximum variance of factor and maximum likelihood estimation was used for rotation of the factor matrix and factor extraction, respectively. The screen plots of the initial operation suggested four components could be extracted to explain the variation of most data (Figure S2). As Table S4 presented, these factors explained over 50% of the total variance, and F1, F2, F3, and F4 accounted for 24.5%, 19.0%, 9.6%, 6.7% in January and 20.1%, 18.4%, 14.5%, 6.0% in July, respectively. 

**Figure 3:**
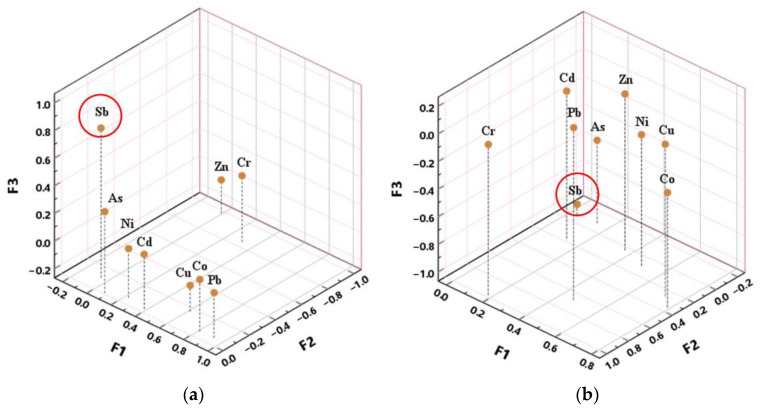
Three-dimensional scatter plots of the first 3 factors’ score coefficients in January (**a**) and July (**b**), 2020. Antimony (Sb) was highlighted for its unique position in factor analysis.

In addition, a similar conclusion was drawn by analyzing the factor score coefficient matrix of FA listed in Table S5 that Sb had a close relationship with F3 for the absolute values of Sb’s coefficient were still highest among the nine coefficients in both periods. Furthermore, the three-dimensional scatter plots of the first three factors’ score coefficients in 2 months were plotted in Figure 3 to visually demonstrate the large gap between the contribution of Sb’s coefficient and that of other PTEs in the F3 column. 

### 3.5. PTEs Concentration in Water Directly Collected from Drainage Outlets and Spatial Distribution of Sb Downstream

**Figure 4:**
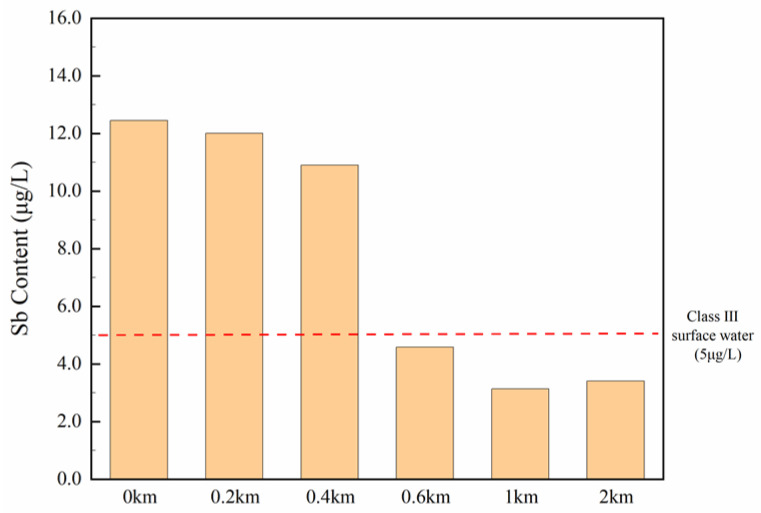
Sb concentration in sampling sites near a drainage outlet of textile wastewater treatment plant (sites 1–1 to 1–5 illustrated in Figure 1c).

The concentration of PTEs in drainage water collected directly from the outlet to the water was measured. To preliminarily study the horizontal diffusion of Sb downstream, sampling site 1 adjacent to the outlet of a textile enterprise was considered as the initial site, then sampling sites 1–1 (200 m), 1–2 (400 m), 1–3 (600 m), 1–4 (1 km) and 1–5 (2 km) located at the downstream river were selected, according to the straight-line distance between the outlet and sampling sites, to collect the surface water samples. As shown in the column chart (Figure 4), Sb concentrations decreased with increasing distance expectedly, from the perspective of the overall trend. Nonetheless, Sb concentrations were not below 5 μg/L (Class Ⅲ threshold) until the distance increased to 600 m, suggesting wastewater discharged from the textile enterprises was able to affect surface water within 400 m away from the outlet at least, which may cause point source pollution. Therefore, it can be concluded that surface water influenced by textile wastewater from outlets was conducted to verify that wastewater from textile enterprises contains relatively high Sb content normally and has an influence on the surface water quality.

The PTEs concentration in drainage water from all 14 sampling sites were shown in Table S6. Concentrations of Sb were larger than the standard limit at 35.71% of sampling sites, indicating that some textile enterprises discharged the wastewater meeting the demand of textile and dying industrial effluent standard limits (GB 4287-2012) rather than that of surface water Class Ⅲ threshold. In contrast, the concentration of other PTEs was not significantly higher than that of the surface water. There was only one water sample that showed a relatively high Ni concentration that exceeded the class Ⅲ threshold of surface water standards, while the others all reached standard limits. A potential explanation of the high Ni concentration in this sample is the contribution from manufacturing workshops and stores associated with the usage of Ni, including hardware store (355 m from the sampling site), decoration material store (450 m), and electric wire workshop (504 m). As nickel is used for the production of stainless steel and other alloys with high corrosion and temperature resistance [38], the above places are likely to be the source of Ni in that particular location. There was no significance in analyzing Cd concentrations’ statistical values because they were collectively lower than the detection limit. 

## 4. Discussion

### 4.1. Physicochemical Characteristics of Surface Water and Potential Toxic Element Concentrations in Dry Period and Wet Period

As the results of physicochemical indicators and PTE contents show in Table 1 and Table 2, there was generally a gap between these means and CVs in the dry period and those in wet periods, which may result from the difference in the numbers and distribution of sampling site in two periods, decreased industrial and anthropogenic activities for the arrival of Chinese Spring Festival vocation, and the novel coronavirus epidemic in January, as well as the seasonal change in hydrodynamic conditions.

The absolute deviation of Sb’s CVs was lowest at 7.48%, indicating the aforementioned factors had the least impact on the dispersion of Sb content among these PTEs. Compared with the abundance of Sb in natural fresh water at the concentration of 1 μg/L at most [39], over 70% of the determined concentrations in all water samples of two periods was absolutely beyond the reference value. It can be inferred from this information that the Sb in the surface water was possibly derived from the point source rather than the natural source.

According to the results of Spearman correlation analysis, only Sb contents were significantly correlated with SPC (*r* = 0.525 in January, 0.589 in July) and TDS (*r* = 0.474 in January, 0.591 in July) at *p* < 0.01, suggesting Sb in the surface water of study area mainly exists in the form of dissolved matter and metal ions. It was reported that Sb (Ⅲ), Sb (Ⅴ), and organoantimony compounds are the three species of antimony in the aqueous solution [25]. In addition, the study on Sb compounds in textile effluent found that Sb (Ⅴ) is the most specie in textile effluent, while Sb (Ⅲ) which is considered to be more toxic than Sb (Ⅴ) is rare under aerobic conditions in textile processing [40]. Therefore, the significant correlation can explain that Sb (Ⅴ) generated from the wet processing of the textile industry on fabrics rather than the natural factors such as soil erosion had been transferred to surface water by wastewater emission.

### 4.2. Impact of Textile Industries on Sb Distribution in Surface Water

**Figure 5:**
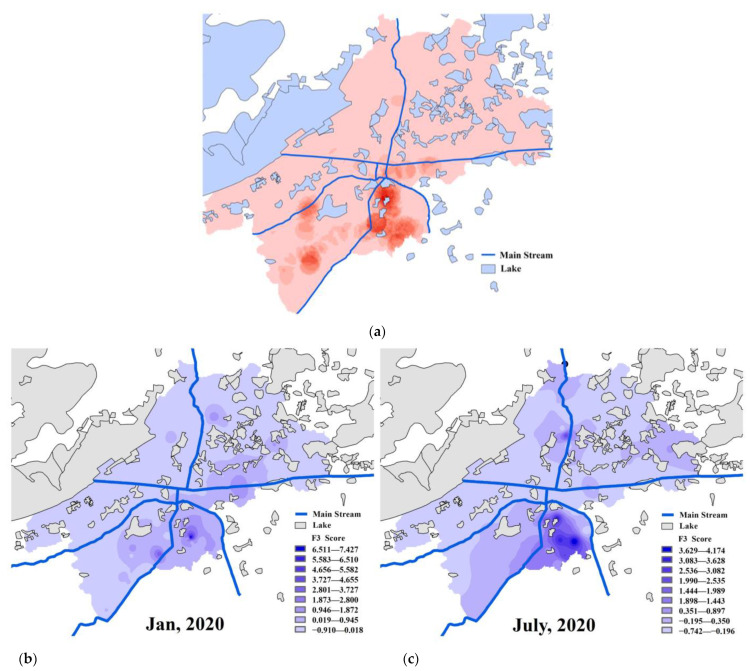
The density distribution of textile enterprises in 2019 (**a**); comparison among the inverse distance interpolation analysis of F3 factor scores in study area in January (**b**) and in July (**c**).

As it has been shown in Figure S1c, the values of normalized Sb contents over 0.5 were concentrated in the water bodies in the southeastern part of the study area, which was located in the region between Jinghang Canal and Sujia Canal, and most large textile enterprises (Figure 5a) were densely distributed in this area. This correspondence does not exist for the other elements analyzed (Figure S1). On the basis of this fact, textile wastewater emission could be regarded as one of the factors influencing the Sb content in nearby surface water.

The results of FA on nine PTEs (Tables S4 and S5) demonstrated that the absolute values of Sb’s loading coefficient and factor score coefficient on F3 were highest in both periods, and the absolute values of these PTE’s loading coefficients were below 0.8 except for Sb. Additionally, F3 score distribution in the study area shown in Figure 5b,c indicated that the darker color represents higher F3 scores, the darker colors were concentrated in the southeastern area and lighter colors in other areas. This is in accordance with the distribution of Sb content and textile enterprise density, which was adequate to suggest that the component represented by F3 was closely correlated with the textile industry. Significantly high Sb in treated wastewater sampled from drainage outlets of textile factories also supported this finding (Table S6). According to the regular fabric treatments, the wet processing of the textile industry including de-sizing, scouring, bleaching, mercerizing, dyeing, and finishing accounts for a large proportion of water consumption in textile production [41]. Diantimony trioxide (Sb_2_O_3_) is often used as a catalyst in the fabrication of polyethylene terephthalate (PET) for improving the wash fastness property and as a flame retardant in the finishing process of textiles such as woolen fabrics for adding the fireproof property [42,43,44]. Furthermore, Sb is mainly released from the textile effluent of de-sizing, mercerizing, and dyeing, because the breaking of the ester bond caused by hydrolysis occurs on the surface of PET fabrics during these processes [10]. In addition, Sb in textile wastewater cannot be effectively removed by traditional processes such as biochemical treatment and filtration treatment [19,45]. Therefore, the textile industries are the source of Sb in nearby aquatic environments. Referring to the aforementioned context, the significant conclusion obtained is that the water drained from textile industries, even after treatment, was the main reason for the significantly high Sb content in the surface water in the area.

### 4.3. Contribution of Sb to the Heavy Metal Pollution Risk Assessment

Despite of huge difference between *PLI* and the other two methods, the fact that cannot be ignored is that the contribution of Sb. Its unit weight and sub-index on *HPI* and *CF* on *PLI* and *NPI* in two months were both higher than other PTEs (Figure 2), indicating the pollution risk of Sb rather than other PTEs in the Wujiang area should be paid more attention. In addition, according to the monitor data of PTE concentrations in the water adjacent to outlets of textile enterprises, the proportion of Sb content over the Class Ⅲ surface water standard limit was much higher than that in the surface water, which means wastewater emission of these textile enterprises will not give rise to the serious heavy metal pollution incident but result in the hidden risk to drinking water quality and ecological environment. Moreover, the content of Sb in water decreased downstream, which means the high concentration of Sb may have a certain connection with the drainage outlets of textile enterprises.

## 5. Conclusions

This study mainly shows the distribution and variation of Sb and other 8 PTEs in the surface water of Wujiang County in the wet period (January) and dry period (July). The eco-risk and source of Sb was distinct from the other elements. The absolute deviation of Sb’s CVs in two periods was significantly lower than other eight PTEs, suggesting seasonal change had the least influence on Sb contents. The interpolation analysis of PTE content in study area demonstrated their distribution varied significantly with the seasonal change except for Sb, while Sb content in two periods was both concentrated in the southeastern part of study area. Significant positive correlation between Sb and water properties including SPC and TDS indicated that Sb mainly exists in the binding with dissolved matter. Factor analysis on the whole dataset of PTE showed Sb was dominated in F3 and was distributed in the area with dense textile factories. Therefore, it can be inferred that the textile wastewater emission is the main reason for the abnormal Sb content in the surface water. The results regarding the heavy metal pollution index (*HPI*), pollution load index (*PLI*), and Nemerow pollution index (*NPI*) showed the Sb contributed most on potential eco-risk to local aquatic environment. This shows that current regulation is not adequate to maintain local aquatic environment quality. Although the standard on wastewater emission of textile industry had been revised and added the emission limit of total Sb concentration with 0.1 mg/L in 2012 (GB 4287-2012), it would be necessary to strengthen administrative supervision on local textile enterprises and elevate the local standard of textile wastewater emission. 

## Figures and Tables

**Table 1:** Physicochemical property indicators of surface water samples in study area during two periods.

Physicochemical Property	Dry Period (January, 2020; *n* = 154)				Wet Period (July, 2020; *n* = 74)			
	Max	Min	Mean	CV * (%)	Max	Min	Mean	CV * (%)
Temperature (°C)	11.29	7.73	8.81	7.38	32.85	26.19	28.57	5.59
pH	8.03	6.94	7.43	2.83	7.35	6.19	7.05	3.98
DO (mg/L)	12.56	4.18	9.09	20.06	16.09	0.86	6.48	50.48
SPC (μs/cm)	995.00	361.40	566.19	19.78	838.10	197.10	387.40	33.78
TDS (mg/L)	979.49	42.15	370.87	29.45	545.00	128.00	251.82	33.77
Turbidity (NTU)	240.30	2.38	53.79	92.75	59.70	3.40	18.45	84.73
COD (mg/L)	49.50	0.10	19.89	44.53	77.60	10.70	31.66	37.73
fDOM (RFU)	445.52	2.75	102.82	70.06	26.39	4.67	11.66	42.29
Chl (μg/L)	76.05	0.46	7.02	144.29	43.98	1.76	11.83	88.81

* CV, coefficient of variance.

**Table 2:** Concentrations of nine PTEs in the surface water samples of study area during two periods (μg/L).

Potential Toxic Elements	Dry Period (January, 2020; *n* = 154)				Wet Period (July, 2020; *n* = 74)			
	Max	Min	Mean	CV ^1^ (%)	Max	Min	Mean	CV ^1^ (%)
Cr	16.30	N ^2^	1.17	151.13	5.40	N ^2^	0.82	110.47
Ni	33.21	0.49	2.89	107.31	10.62	0.82	3.04	58.71
Cu	44.67	N	5.78	120.03	7.75	0.60	2.70	59.63
Zn	1245.05	N	24.68	433.74	183.20	3.52	27.69	110.86
Cd	0.55	N	0.10	98.75	0.17	N	0.08	52.78
Pb	22.48	N	2.42	121.44	5.88	0.21	1.49	69.90
Co	4.27	0.06	0.52	106.56	1.91	0.06	0.48	66.74
As	29.15	0.26	1.65	140.46	7.06	1.37	2.43	43.64
Sb	21.37	0.48	2.26	107.19	7.28	0.49	1.48	99.71

^1^ CV, coefficient of variance. ^2^ N, element concentration lower than detection limit.

## Data Availability

Data cannot be made publicly available, and readers should contact the corresponding author for details.

## Supplementary Materials

The following supporting information can be downloaded at: https://www.mdpi.com/article/10.3390/ijerph20043600/s1, Figure S1: (a) Spatial and temporal distribution of Cr, Ni, Cu and Zn contents in the surface water of study area. The data has been normalized into the same interval scale with [0,1] for better presentation on a contour plot, (b) Spatial and temporal distribution of Cd, Pb, Co and As contents in the surface water of study area. The data has been normalized into the same interval scale with [0,1] for better presentation on a contour plot, (c) Spatial and temporal distribution of Sb contents in the surface water of study area. The data has been normalized into the same interval scale with [0,1] for better presentation on a contour plot; Figure S2: Screen plots of factors obtained after the initial operation in January and July, 2020; Table S1: Details of water quality evaluation methods used in this study; Table S2: Spearman correlation among metals and 4 physicochemical indicators in January; Table S3: Spearman correlation among metals and 4 physicochemical indicators in July; Table S4: Loading coefficients after rotation of factor matrix and variance explanation in two periods; Table S5: Factor score coefficients in two periods; Table S6: Trace metal concentrations in 14 water samples (μg/L).
