# Supplementary material for: Impact of Textile Industries on Surface Water Contamination by Sb and Other Potential Toxic Elements: A Case Study in Taihu Lake Basin, China
Source: Int J Environ Res Public Health. 2023 Feb 17;20(4):3600. doi: 10.3390/ijerph20043600 (PMC9963225; doi:10.3390/ijerph20043600)
Supplement: Supplementary file 1 [file ijerph-20-03600-s001.zip › ijerph-2203257-supplementary.pdf]

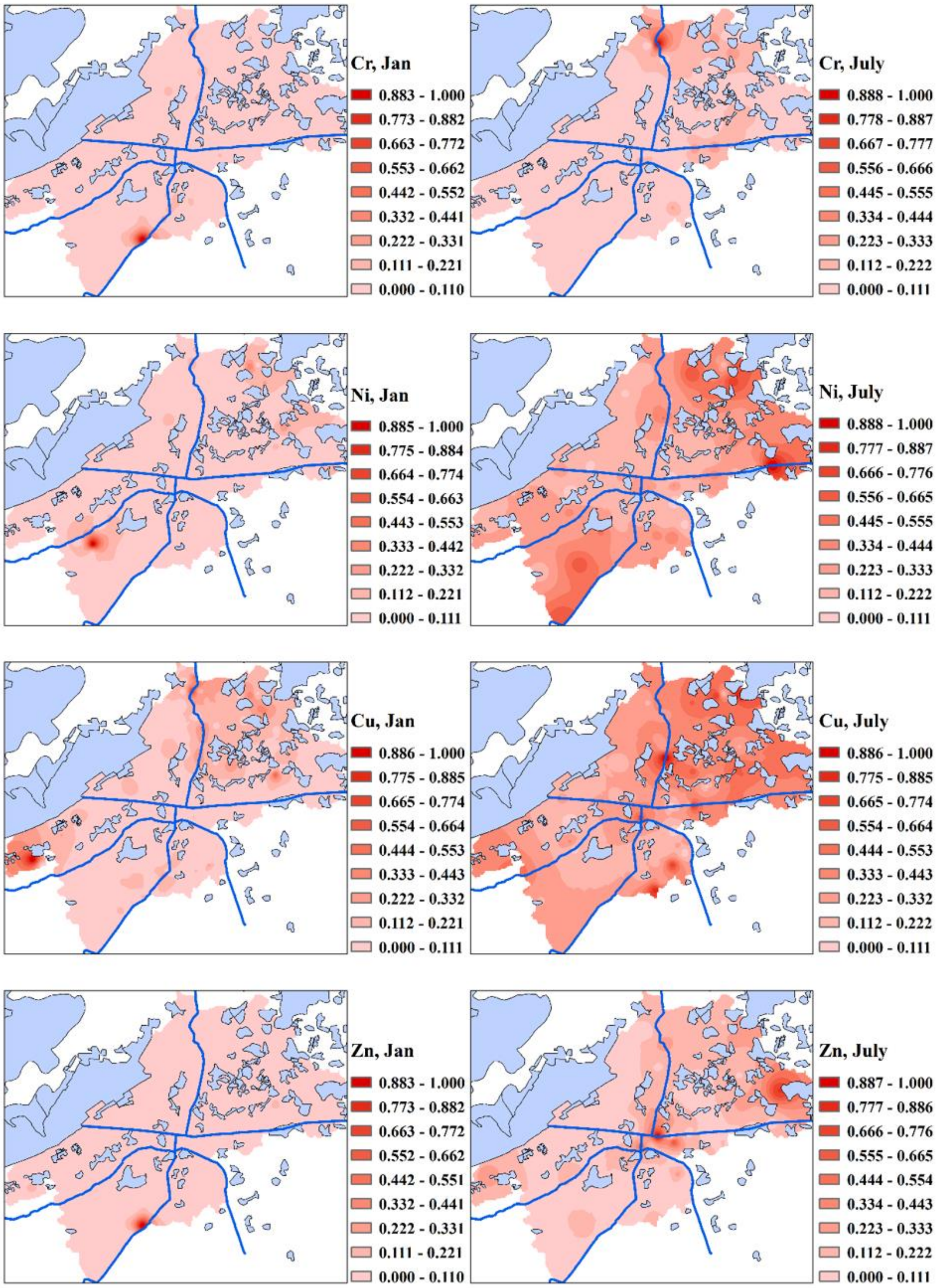

Figure S1. (**a**) Spatial and temporal distribution of Cr, Ni, Cu and Zn contents in the surface water of study area. The data has been normalized into the same interval scale with [0,1] for better presentation on a contour plot.

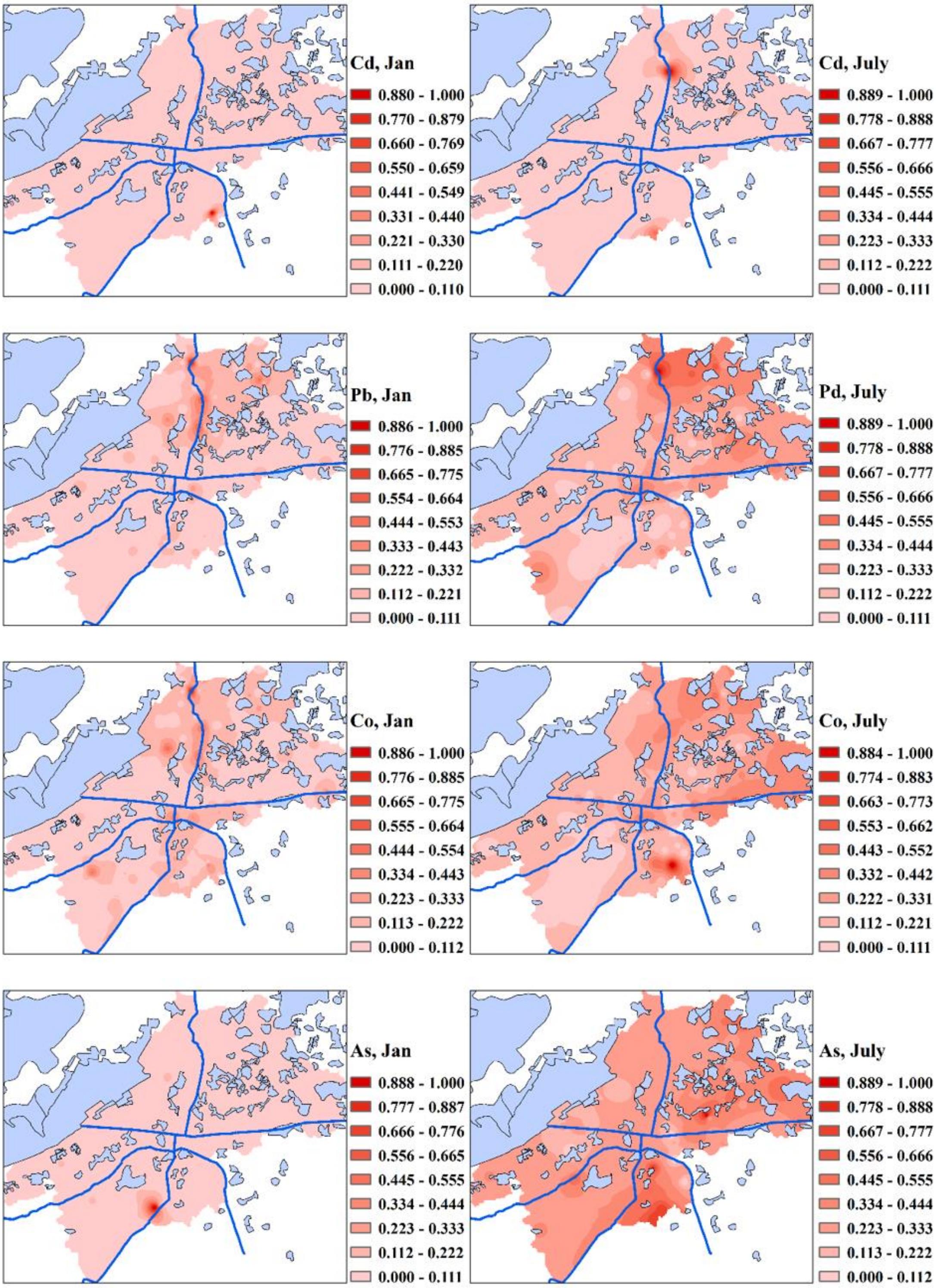

Figure S1. (**b**) Spatial and temporal distribution of Cd, Pb, Co and As contents in the surface water of study area. The data has been normalized into the same interval scale with [0,1] for better presentation on a contour plot.

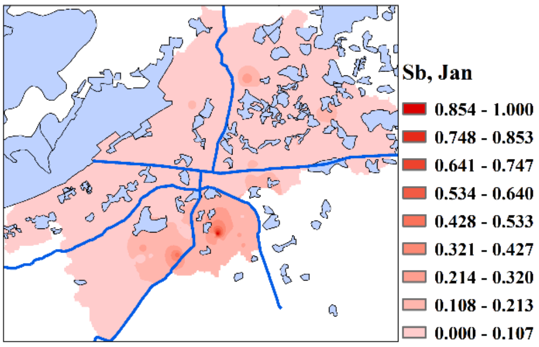

Figure S1. (**c**) Spatial and temporal distribution of Sb contents in the surface water of study area. The data has been normalized into the same interval scale with [0,1] for better presentation on a contour plot.

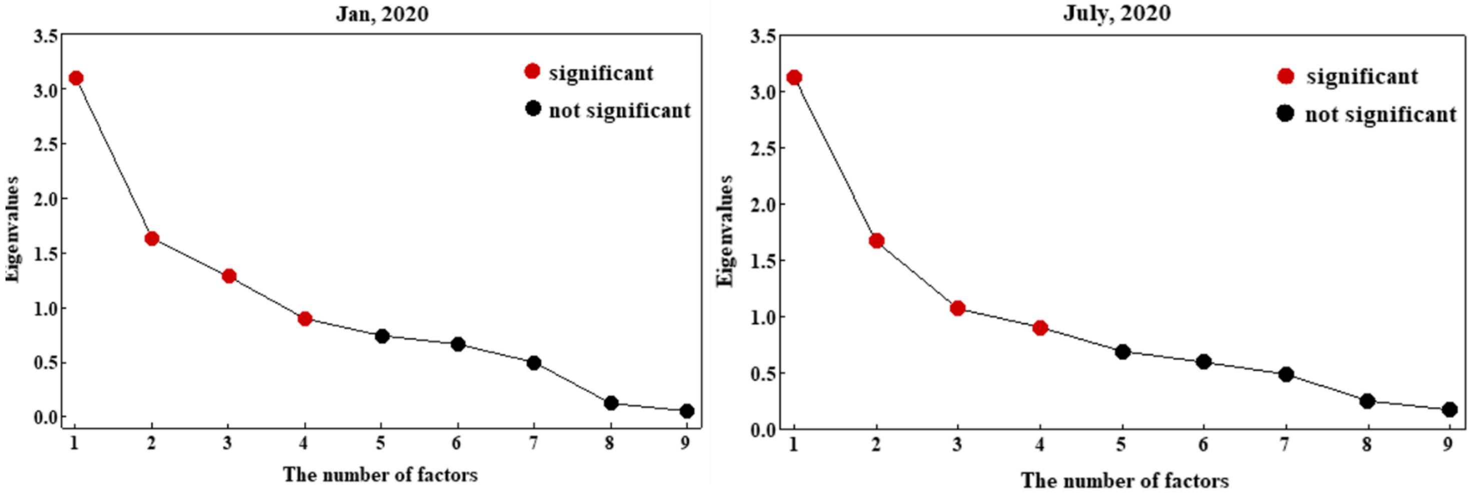

Figure S2. Screen plots of factors obtained after the initial operation in January and July, 2020

Table S1. Details of water quality evaluation methods used in this study.

Method	Pollution level	Classification	Value	References
Heavy metal pollution index (HPI)	Slight	Ⅰ	HPI < 15	[30]
	Moderate	Ⅱ	15≤HPI≤30	
	Moderate-to-heavy	Ⅲ	30≤HPI≤100	
	Heavy	Ⅳ	HPI > 100	
Pollution load index (PLI)	Baseline Level	/	0 < PLI≤1	[35]
	Polluted	/	PLI > 1	
Nemerow pollution index (NPI)	Unpolluted	Ⅰ	NPI < 0.7	[37]
	Slight	Ⅱ	0.7≤NPI≤1	
	Moderate	Ⅲ	1≤NPI≤2	
	Heavy	Ⅳ	NPI > 2	

Table S2. Spearman correlation among metals and 4 physicochemical indicators in January

	Cr	Ni	Cu	Zn	Cd	Pb	Co	As	Sb	SPC	TDS	NTU	COD
Cr	1.000												
Ni	0.798**	1.000											
Cu	-0.110	-0.131	1.000										
Zn	0.369**	0.316**	0.380**	1.000									
Cd	0.210	0.223	0.439**	0.460**	1.000								
Pb	0.245*	0.131	0.779**	0.466**	0.420**	1.000							
Co	0.714**	0.756**	0.137	0.549**	0.265*	0.460**	1.000						
As	0.322**	0.398**	0.170	0.512**	0.232*	0.343**	0.549**	1.000					
Sb	0.475**	0.568**	-0.122	0.073	0.126	0.122	0.412**	0.273*	1.000				
SPC	0.133	0.421**	0.026	-0.034	0.071	-0.051	0.245*	0.197	0.525**	1.000			
TDS	0.072	0.377**	0.009	-0.095	-0.014	-0.057	0.236*	0.207	0.474**	0.930**	1.000		
NTU	0.570**	0.399**	0.045	0.428**	0.182	0.421**	0.612**	0.352**	0.148	-0.338**	-0.276*	1.000	
COD	-0.105	-0.100	0.102	-0.137	-0.057	0.012	0.091	0.020	0.041	0.222	0.296*	-0.082	1.000

* Significant level at $p < 0.05$
** Significant level at $p < 0.01$

Table S3. Spearman correlation among metals and 4 physicochemical indicators in July.

	**Cr**	**Ni**	**Cu**	**Zn**	**Cd**	**Pb**	**Co**	**As**	**Sb**	**SPC**	**TDS**	**NTU**	**COD**
Cr	1.000												
Ni	0.538**	1.000											
Cu	0.497**	0.588**	1.000										
Zn	0.380*	0.739**	0.607**	1.000									
Cd	0.047	0.114	0.231	0.096	1.000								
Pb	0.641**	0.564**	0.514**	0.655**	0.108	1.000							
Co	0.628**	0.619**	0.593**	0.509**	0.156	0.663**	1.000						
As	0.112	0.183	0.071	0.193	0.352*	0.069	0.187	1.000					
Sb	-0.072	0.222	-0.029	0.223	0.277	-0.056	0.129	0.583**	1.000				
SPC	-0.085	0.005	-0.117	-0.093	0.195	-0.354*	0.080	0.679**	0.589**	1.000			
TDS	-0.089	0.007	-0.120	-0.092	0.195	-0.353*	0.077	0.683**	0.591**	0.990**	1.000		
NTU	0.506**	0.322*	0.219	0.099	-0.018	0.494**	0.496**	-0.239	-0.341*	-0.277	-0.279	1.000	
COD	-0.012	0.071	0.259	0.181	0.350*	0.117	0.287	0.516**	0.237	0.362*	0.364*	-0.126	1.000

* Significant level at $p < 0.05$

** Significant level at $p < 0.01$

Table S4. Loading coefficients after rotation of factor matrix and variance explanation in two periods.

Trace Metals	Non-flood Period (Jan, 2020; n=154)				Flood Period (July, 2020; n=74)			
	F1	F2	F3	F4	F1	F2	F3	F4
Cr	0.312	-0.842	0.215	-0.136	0.180	0.981	0.024	-0.063
Ni	0.227	-0.055	0.090	-0.580	0.494	0.099	-0.118	-0.164
Cu	0.615	-0.166	-0.076	-0.068	0.724	0.264	0.034	-0.174
Zn	-0.005	-0.977	-0.023	0.008	0.363	0.032	0.064	-0.176
Cd	0.384	-0.028	0.130	-0.126	0.126	0.172	0.001	-0.268
Pb	0.927	-0.060	0.061	-0.098	0.464	0.671	0.180	-0.155
Co	0.812	-0.057	0.110	-0.466	0.789	0.351	-0.235	0.164
As	0.082	-0.007	0.324	-0.064	0.139	-0.086	-0.467	-0.565
Sb	-0.055	-0.104	0.818	-0.007	0.036	-0.074	-0.989	-0.077
Eigenvalues	2.203	1.713	0.867	0.606	1.808	1.659	1.304	0.540
% of variance	24.5	19.0	9.6	6.7	20.1	18.4	14.5	6.0
Cumulative %	24.5	43.5	53.1	59.8	20.1	38.5	53.0	59.0

Table S5. Factor score coefficients in two periods.

Trace Metals	Non-flood Period (Jan, 2020; n=154)				Flood Period (July, 2020; n=74)			
	F1	F2	F3	F4	F1	F2	F3	F4
Cr	0.042	-0.149	0.227	-0.051	-0.450	1.102	-0.097	-0.037
Ni	-0.078	-0.001	-0.003	-0.328	0.109	-0.027	0.013	-0.098
Cu	0.102	-0.007	-0.060	0.085	0.294	-0.068	0.034	-0.200
Zn	-0.092	-0.854	-0.259	0.051	0.071	-0.020	0.013	-0.100
Cd	0.030	0.002	0.034	0.000	0.018	-0.012	0.012	-0.134
Pb	0.683	0.022	-0.002	0.640	0.206	-0.052	0.034	-0.210
Co	0.220	0.024	0.002	-0.851	0.667	-0.087	-0.019	0.543
As	-0.005	0.002	0.104	-0.005	0.050	-0.046	0.041	-0.557
Sb	-0.061	0.001	0.735	0.074	-0.144	0.055	-1.009	0.054

Table S6. Trace metal concentrations in 14 water samples (μg/L).

Trace Metals	Cr	Ni	Cu	Zn	Pb	Co	As	Sb
Max	6.44	489.63	65.38	126.09	3.41	183.50	12.77	20.95
Min	2.02	4.74	0.87	0.23	0.11	0.25	2.10	2.26
Mean	3.54	44.04	6.99	15.71	0.70	15.84	5.09	7.16
CV%	41.15	291.61	241.38	204.88	173.22	318.50	56.78	75.99
